# Oral hygiene practices and dental caries prevalence among 12 & 15 years school children in Ambala, Haryana -A cross-sectional study

**DOI:** 10.4317/jced.52303

**Published:** 2015-07-01

**Authors:** Richa Goel, Archita Vedi, Koratagere-Lingappa Veeresha, Girish-Malleshappa Sogi, Ramandeep-Singh Gambhir

**Affiliations:** 1 BDS, MDS, Senior Lecturer, Dept. of Public Health Dentistry, MM University, Mullana, Ambala; 2 BDS, MDS, Professor, Dept. of Public Health Dentistry, MM University, Mullana, Ambala; 3 BDS, MDS, Professor and Head, Dept. of Public Health Dentistry, MM University, Mullana, Ambala; 4 BDS, MDS, MPH, Reader, Dept. of Public Health Dentistry, Gian Sagar Dental College and Hospital, Rajpura, Punjab

## Abstract

**Background:**

Dental caries and gum disease are a major public health problem predominantly affecting children worldwide. Therefore the present study was conducted to assess oral hygiene practices, prevalence of dental caries and periodontal diseases among 12 and 15 years old high school children in public and government schools of Ambala district, Haryana and to provide data for planning and evaluation of oral health care promotion programs.

**Material and Methods:**

A cross-sectional descriptive survey of 12 and 15 years old children in government and private schools was conducted in Ambala, Haryana. A sample of 992 school children was selected by a two-stage cluster sampling method. Dental caries and periodontal health was examined using WHO standard criteria. The student’s t-test and one way ANOVA were used for statistical analysis.

**Results:**

The prevalence of dental caries was 34.3% and 46.5% at 12 and 15 years respectively. Mean DMFT in 12 years was 0.82 and in 15 years it was 1.26. More than 90% of subjects in both the age groups were using tooth brush to clean their teeth. Toothpaste was used by 96.7% (228) of subjects belonging to 12 year age group and 93% (703) of subjects who were of 15 years of age. Gingival bleeding was found in majority of subjects.

**Conclusions:**

The overall prevalence of dental caries was low but the prevalence of gingivitis was quite high. The observations indicate the need for a school oral health promotion programme to sustain the healthy practices in this growing population.

** Key words:**Dental caries, periodontitis, ambala, school children, oral hygiene practices.

## Introduction

Dental caries because of its ubiquitous nature remains one of the most prevalent afflictions of mankind (1). It continues to be a major public health problem predominantly affecting children in spite of its preventable nature and creditable scientific advances in its treatment modalities. It is cumulative process which if not intervened in the incipient stages ultimately leads to tooth loss. It is also the most common oral disease responsible for the absenteeism from schools and loss of working hours (2). Children who suffer from poor oral health are 12 times more likely to have more restricted activity days including missing school than those who do not (3).

Children are very important part of a country’s demography and their health influences the future of nation. Schools are micro-cosms of the larger community and provide the ideal setting for integrated health promotion (3). School age is regarded as the phase of child hood during which a child acquires the knowledge of the norms and values of a society and emerges as a contributing member to the community. Hence it is an influential stage in people’s life when lifelong sustainable oral health related behaviors, beliefs and attitudes can be established with longer lasting impact. Moreover, the messages can be reinforced throughout the school years (4).

At the age of 12 years permanent teeth (except 3rd molars) would have erupted and by 15 years age these teeth are exposed to the oral environment for almost 3 years. WHO has recommends both these ages as the index ages for oral health assessment (5).

There is plethora of literature available on prevalence of dental caries and periodontal conditions in various population groups in different parts of our country. However, there is scant information on the oral health status including dental caries and periodontal disease prevalence among school children in various district of Haryana. The following study aims at assessing the oral hygiene practices, prevalence of dental caries and periodontal diseases among the 12 & 15 years old school students in Ambala district of Haryana state. It will help establish a reliable baseline data for planning and development of national or regional oral health programs.

## Material and Methods

-Ethics and informed consent 

Ambala is one of the 21 districts of Haryana state in India. The entire district is divided into 6 blocks for administrative purpose. The study protocol was reviewed and ethical clearance was granted by Institutional Review Board, MM University, Mullana. An official permission and list of all public and government middle and high schools were obtained from the District Education Officer, Ambala and also from the principals of the respective schools. After explaining the purpose and details of the study, a written informed consent was obtained from parents of all children who fulfilled the eligibility criteria and were willing to participate in the survey.

-Sampling technique

The sample frame consisted of middle and high schools (public and government) in Ambala, and the study sample was recruited by a two-stage cluster sampling technique. Both public and government schools from each block were randomly selected to obtain the desired sample size. The sample size was calculated on the basis of dental caries and periodontal disease prevalence reported in the pilot survey. Out of the total number of private (32) and government (58) schools, fifteen public and twenty five government schools were randomly selected. In the second stage, eligible school children were stratified according to age and gender, and randomly selected in proportions to the total number of 12 & 15 years old students enrolled in each school to reach the sample of 992. The final sample included more no. of 15 year children (756) as compared to 12 year children (236) as the corresponding grade of 15 year children had three sections as compared to only one section of corresponding grade in which 12 year children were studying in majority of the schools that were visited.

-Inclusion and Exclusion

School children (males & female) who have completed 12 & 15 years of age and present on the day of examination were included in the study. Children with any systemic disease, on antibiotic therapy in the previous six months and who refused to participate were excluded from the study.

-Calibration and Pilot survey 

Before the commencement of the study, training and intra-examiner calibration was done in the Department of Public Health Dentistry (kappa value=95%). A pilot survey was also carried out among 100 children, from one public and one government school to determine the feasibility of the study. Depending on the prevalence obtained, 95% confidence level and 5% allowable error, the sample size was determined to be 990.

-Data collection and examination

Data collection was carried out by a single investigator in order to simplify the operational process of data collection who was assisted by a recording assistant. A total of 992 subjects were examined. Data regarding general information, oral hygiene practices were obtained through interview and recorded on a pre-structured performa. Clinical examination (type III) included dental caries and periodontal status examination (using CPI probe) using WHO standard criteria as mentioned in the WHO Oral Health Proforma, 1997 (5). However, radiographs were not used for diagnostic purpose. To reduce the examiner bias, duplicate examination was conducted on 5% (n=100) of the population during course of the study. Referral was forwarded to the parents of the children in need of dental care. After the conclusion of the survey, an oral health education session and tooth brushing demonstration was conducted for all the students and teachers in the school.

-Statistical analysis

The recorded data was compiled and entered in a spread sheet (Microsoft excel 2010) and then exported to data page of SPSS version 15 (SPSS Inc., Chicago, Illinois, USA). The student’s t- test and one way ANOVA were used for analysis. Multivariate logistic regression analysis was performed to assess the effect of various independent variables on prevalence of dental caries. Odds ratios (OR) with 95% CI were also reported. For all test, confidence interval and *p*-value were set at 95% and < 0.05 respectively.

## Results

**Figure 1:**
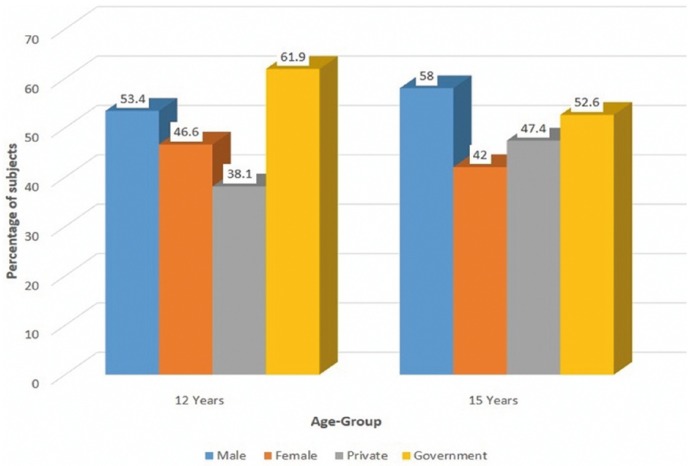
Demographic details of the study population.

A total of 992 subjects were enrolled in the present survey. A total of 236 (23.7%) were of 12 years of age and 756 (76.3%) be-longed to 15 years age-group. 57% (564) were males and 43.1% (428) were females. Percentage of subjects belonging to private and government schools were 45.2% (448) and 54.8% (544) respectively. Detail information regarding age, gender and type of school is depicted in figure 1.

-Oral hygiene practices

More than 90% of subjects in both the age groups were using tooth brush to clean their teeth. Toothpaste was used by 96.7% (228) of subjects belonging to 12 year age group and 93% (703) of subjects who were of 15 years of age. 68.6% (162) of 12 year old subjects and 77.4% (585) of 15 year old subjects were brushing their teeth at least once a day. Tooth brush as a tongue cleaning aid was used by 31.8% (75) of 12 year subjects and 41.4% (313) of subjects belonging to 15 years.  Table 1 provides detailed information on the various oral hygiene practices of subjects belonging to both age-groups. However, no significant difference was obtained regarding oral hygiene practices between both the age groups (*p*>0.05).

**Table 1:**
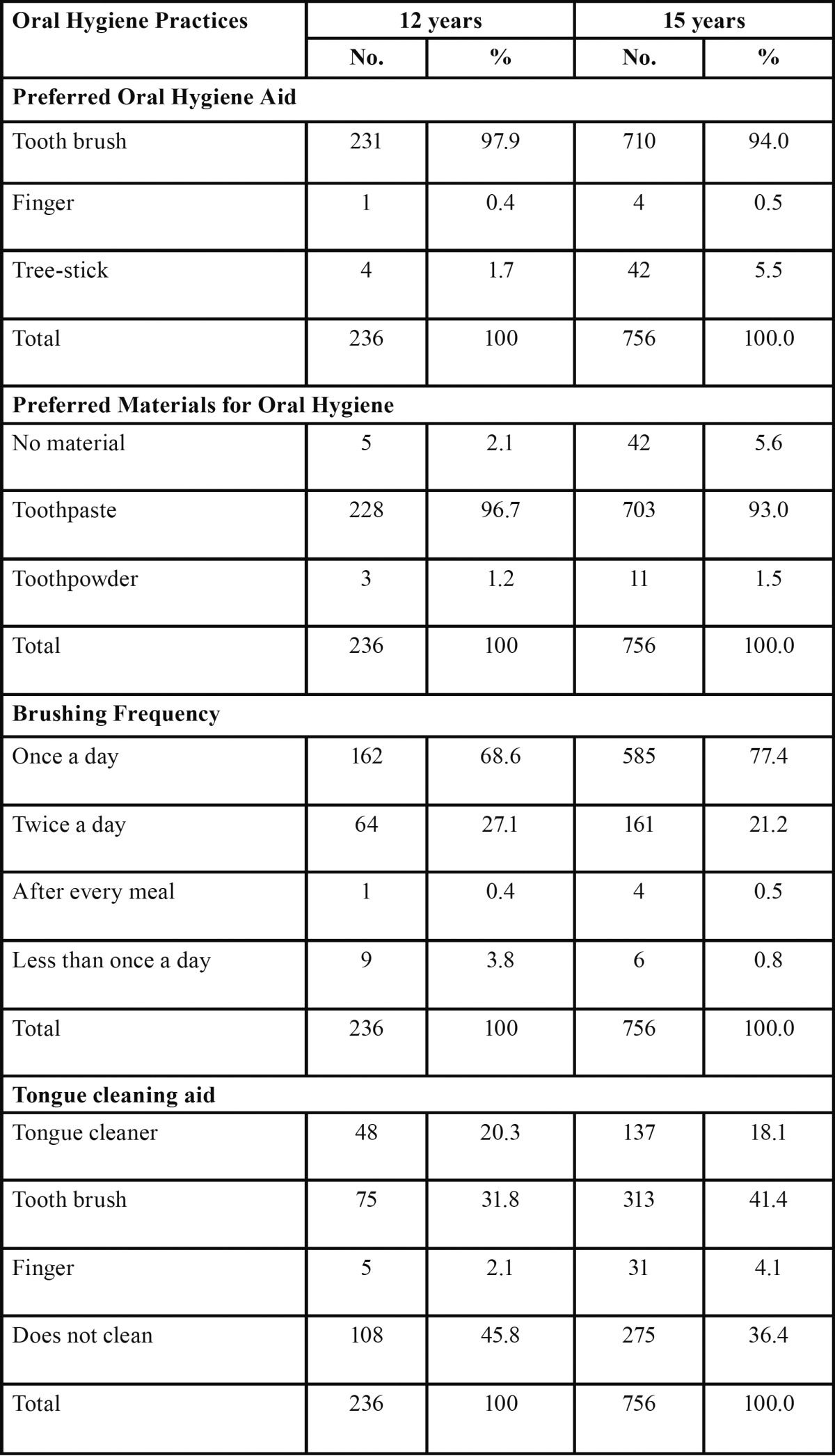
Various oral hygiene practices of subjects of 12 and 15 year age groups.

-Dental caries 

The prevalence of dental caries in 15 years age group was 46.5% and 34.3% in 12 year age-group. The mean DMFT was 1.26 in 15 year age group and 0.82 in 12 year age group ( Table 2).

**Table 2:**
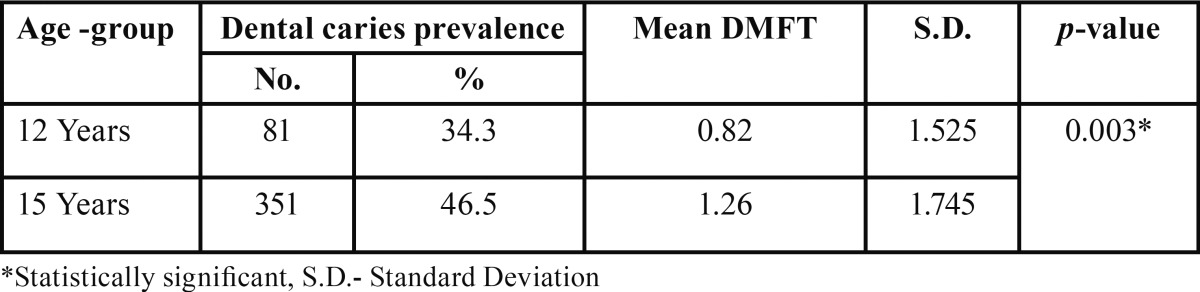
Prevalence of dental caries.

-Periodontal status 

The periodontal status in the study was assessed using the community periodontal index and it was expressed in terms of CPI score.  Table 3 depicts the periodontal status of study subjects belonging to both the age groups. Bleeding (score 1) was the main finding present in majority of the subjects in both the age-groups (69.4% in 12 years and 63.8% in 15 years) followed by calculus (score 2). Very few children had healthy component of gingiva and it was higher at 15 years of age than 12 years. None of the subjects in both the age-groups had a score of 3 and 4 indicative of shallow and deep periodontal pockets respectively.

**Table 3:**
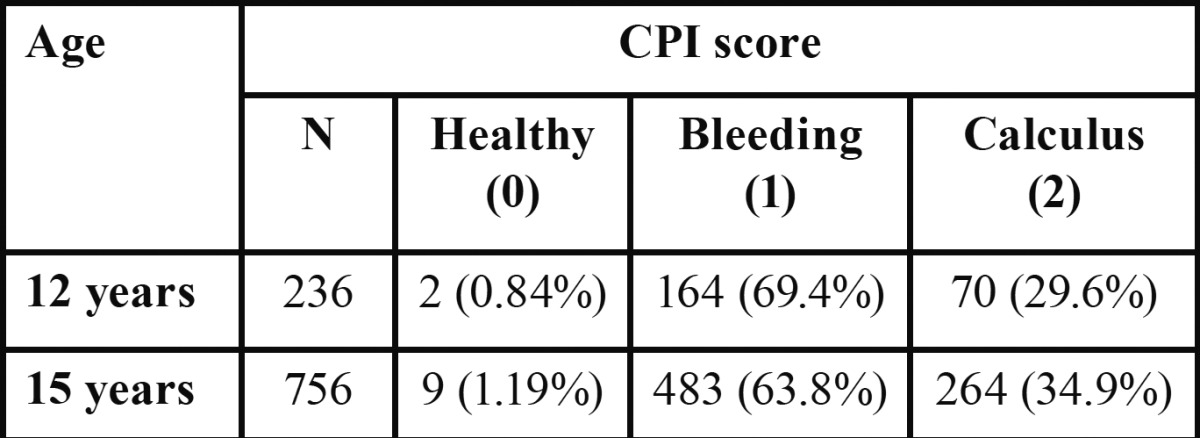
Periodontal status of study population.

-Multiple Logistic Regression Analysis

Multiple logistic regression analysis was performed to assess the effect of various independent variables (age, gender, type of school etc.) on the presence and absence of dental caries. Odds ratios (OR) were also generated ( Table 4). The odds of developing dental caries were 1.45 higher in 15 year age group as compared to subjects of 12 years. Prevalence odds ratios for dental caries were 1.73 times more for female subjects as compared to male subjects, 2.46 times more for those studying in government schools and 2.60 times for those brushing once a day.

**Table 4:**
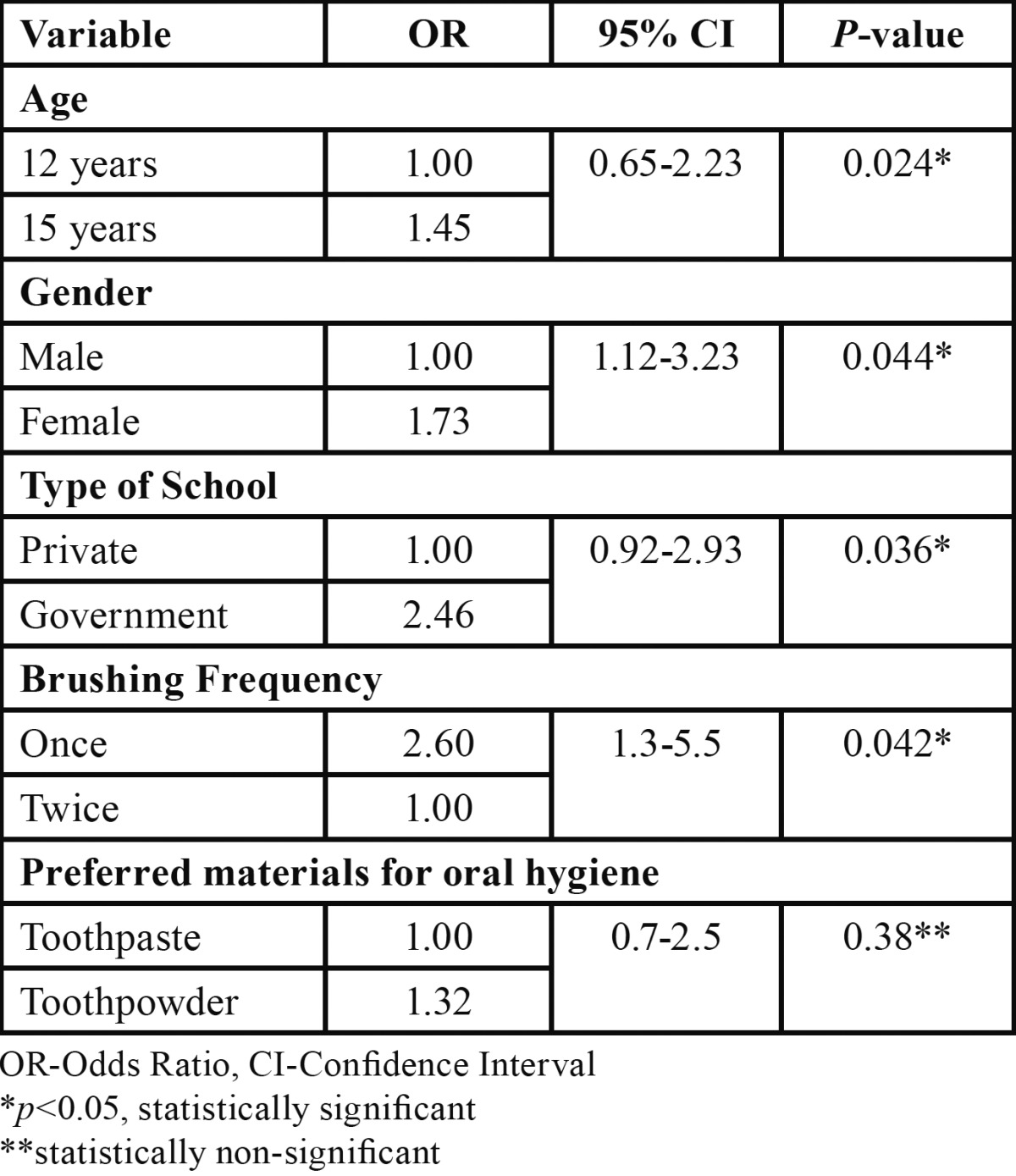
Multiple logistic regression analysis.

## Discussion

Schools provide a platform for the promotion of health and oral health not only for the students, but also for the staff, families, and members of the community as a whole. A total of 992 subjects belonging to both private and government schools were included in the present survey. It was found that number of male subjects were comparatively higher as compared to female subjects in both the age-groups. Though there is an improvement in the state sex ratio in Haryana from 1000:868 (2001) to 1000:877 (2011) and literacy rate has increased to 76.6% (2011) still the overall female participation in the education system is low (6). This gender gap could be due to the reason that in rural areas still the women are not encouraged for school education.

Poor dental hygiene can result in tooth decay, gingivitis, periodontitis, tooth loss, bad breath (halitosis), fungal infection and gum diseases. The use of a toothbrush is the most important measure for oral hygiene (7). Though more than 90% of the study subjects used to brush their once a day, very few subjects were brushing their teeth twice daily which is less as compared to reports of some other study conducted on school children in Lithuania (8). This may be due to the lack of awareness regarding importance of brushing at night or due to peer-influence and lack of parental and professional education. However, number of subjects using tree-stick for cleaning their teeth were much higher (59%) in some other study as compared to the present study (9).

In the present study the prevalence of dental caries was observed at 43.64%. It ranged from 34.3% (12 years) to 46.5% (15 years) which showed an upward trend with increasing age. Some other studies also reported similar prevalence (10,11). However, a higher prevalence was found in a study conducted on school children in Chennai (12). As Ambala lies in a high fluoride belt region, the lower caries prevalence in our study could probably be due to it. On the contrary, a lower caries prevalence was reported in some other study (13). The reason for the higher prevalence of dental caries at 15 years compared to 12 years may be because of the cumulative nature of the dental caries which if untreated is a continuous process which may increase with advancing age.

Gingival bleeding was the main finding which was present in about 70% of children of 12 years and 63% of 15 years. Similar finding was also reported in other studies conducted on school children in Thailand and Chennai (14,15). Conversely, lesser number of subjects reported gingival bleeding in the study reports of Dhar *et al.* (16). The higher proportion of gingival bleeding may be attributed to the oral habits like mouth breathing which is usually associated with inflamed adenoids or upper respiratory tract infections, dental conditions like malocclusion, poor oral hygiene and pubertal changes in girls (17). This thing needs to be further explored.

Multivariate analysis in our showed study that those children who were studying in government schools were more at risk of developing dental caries as compared to subjects studying in private schools. A common feature in majority of Indian government schools is the poor quality of education, with weak infrastructure and inadequate pedagogic attention. These schools have witnessed a decline in their services, and increasingly they are accessed by the poor and the marginalized (18).

There were also limitations of the present study. Since the present study was a field survey therefore radiographs were not used in the identification and diagnosis of dental caries. It is likely, therefore, that the prevalence of dental caries may have been under-estimated. Limited time was given by the school authorities for conducting the survey as students were examined during the school hours, therefore detailed assessment of gingivitis was not done using plaque index or gingival index and was based on CPI score only. It was beyond the scope of the present study to assess the etiological factors predisposing to dental caries and periodontal conditions among 12 and 15 years school children therefore, there is a scope for future study.

## Conclusions

The mean DMFT was 1.26 in 15 year age group and 0.82 in 12 year age group. More than 90% of the students used toothbrush to clean their teeth and were brushing at least once a day. Majority of the children reported gingival bleeding. Oral health promotion through integrated school health programs including oral screening, preventive programs like fluoride mouth rinse etc. and health education of school students should be taken up on regular intervals for educating and creating awareness regarding oral health maintenance. Various professional bodies and existing dental colleges can help the community in this regard.
